# Benzodiazepine use in medical cannabis authorization adult patients from 2013 to 2021: Alberta, Canada

**DOI:** 10.1186/s12889-024-18356-6

**Published:** 2024-03-20

**Authors:** Cerina Dubois, Heidi Fernandes, Mu Lin, Karen J. B. Martins, Jason R. B. Dyck, Scott W. Klarenbach, Lawrence Richer, Ed Jess, John G. Hanlon, Elaine Hyshka, Dean T. Eurich

**Affiliations:** 1 School of Public Health, University of Alberta, 3-300 Edmonton Clinic Health Academy 11405 - 87 Ave Edmonton, AB, T6G 1C9 2E, Edmonton, AB Canada; 2 SPOR (Strategy for Patient Oriented Research) Data Platform, Alberta Health Services, Edmonton, Alberta Canada; 3 Faculty of Medicine & Dentistry, University of Alberta, Edmonton, Alberta Canada; 4 Cardiovascular Research Centre, Department of Pediatrics, Faculty of Medicine and Dentistry, University of Alberta, Edmonton, Alberta Canada; 5 College of Physicians & Surgeons of Alberta, Edmonton, Alberta Canada; 6 St. Michael’s Hospital Department of Anesthesia, University of Toronto, Ontario, Canada; 7 Department of Anaesthesiology and Pain Medicine, University of Toronto, Ontario, Canada

**Keywords:** Medical cannabis, Benzodiazepine, Diazepam equivalence, Anxiety, Epidemiology, Cohort study

## Abstract

**Background:**

Benzodiazepines are a class of medications that are being frequently prescribed in Canada but carry significant risk of harm. There has been increasing clinical interest on the potential “sparing effects” of medical cannabis as one strategy to reduce benzodiazepine use. The objective of this study as to examine the association of medical cannabis authorization with benzodiazepine usage between 2013 and 2021 in Alberta, Canada.

**Methods:**

A propensity score matched cohort study with patients on regular benzodiazepine treatment authorized to use medical cannabis compared to controls who do not have authorization for medical cannabis. A total of 9690 medically authorized cannabis patients were matched to controls. To assess the effect of medical cannabis use on daily average diazepam equivalence (DDE), interrupted time series (ITS) analysis was used to assess the change in the trend of DDE in the 12 months before and 12 months after the authorization of medical cannabis.

**Results:**

Over the follow-up period after medical cannabis authorization, there was no overall change in the DDE use in authorized medical cannabis patients compared to matched controls (− 0.08 DDE, 95% CI: − 0.41 to 0.24). Likewise, the sensitivity analysis showed that, among patients consuming ≤5 mg baseline DDE, there was no change immediately after medical cannabis authorization compared to controls (level change, − 0.04 DDE, 95% CI: − 0.12 to 0.03) per patient as well as in the month-to-month trend change (0.002 DDE, 95% CI: − 0.009 to 0.12) per patient was noted.

**Conclusions:**

This short-term study found that medical cannabis authorization had minimal effects on benzodiazepine use. Our findings may contribute ongoing evidence for clinicians regarding the potential impact of medical cannabis to reduce benzodiazepine use.

**Highlights:**

• Medical cannabis authorization had little to no effect on benzodiazepine usage among patients prescribed regular benzodiazepine treatment in Alberta, Canada.

• Further clinical research is needed to investigate the potential impact of medical cannabis as an alternative to benzodiazepine medication.

## Background

Benzodiazepines are anxiolytic medications which are known for their sedative properties in the treatment of anxiety [1]. When first introduced on the market, clinicians enthusiastically prescribed benzodiazepines, quickly becoming one of the most widely prescribed medications [2]. Benzodiazepines have a relaxing or calming effect, which is beneficial in the treatment of anxiety. Benzodiazepines can also relieve severe emotional distress, such as panic attacks. Alongside its benefits, also comes a list of side effects associated with the medication [3]. The most reported side effect is drowsiness and hence they are often used as sleep aids. Other side effects include impaired coordination, slurred speech, confusion, disorientation, dizziness, decreased blood pressure and respiratory rate, and memory problems [4]. Long-term use of the medication, particularly in the elderly population, has been associated with cognitive impairment and increased risk of falls [5].

To date, an increasing number of benzodiazepines are being misused by patients and prescription percentages have aligned with patients’ increased risk of dependence on this medication [6]. While over 1 million benzodiazepine prescriptions were dispensed in Alberta in 2021 alone [7]. Prescribing are seemingly decreasing (4% of Albertans received a benzodiazepine or Z-drug [zopiclone, zaleplon, and zolpidem] prescription in 2021 compared to 9.1% in 2016 – a drop of almost one fifth), in which medical cannabis use may be playing a role in circumventing benzodiazepine usage [7]. Nevertheless, benzodiazepine prescribing continues to be high in Alberta.

Among the top reasons for medical cannabis use has been an increasing clinical interest in its “sparing effect” on reducing the use of other medications, specifically benzodiazepines (as well as opioids) [8]. Literature that supports the use of medical cannabis as a substitution for benzodiazepines is currently low; and studies assessing medical cannabis’ association with anxiety report inconclusive or mixed results [9–11]. One small randomized controlled trial (RCT) investigating 24 patients with generalized social anxiety disorder showed a reduction in public speaking anxiety with the use of cannabidiol (CBD), a therapeutic component of cannabis [12]. Conversely, there are concerns cannabis can actually increase anxiety levels. One study reported cannabis use increased the risk for more severe anxiety symptoms [13], however, a meta-analysis showed that cannabis was only a minor risk factor for increased anxiety [14]. This lack of consensus of cannabis use for anxiety may also be attributed to the inconsistent nature of the current available literature. Previous studies either tend to strongly focus on the negative effects of cannabis and anxiety (rather than searching for how cannabis may improve anxiety) [15–18], consist of age-specific and small cohort sizes [13, 19–23], did not differentiate cannabis from other illicit substances [24, 25], or cannot accurately distinguish cannabis for medical purposes versus recreational where non-medical use which often occur in the context of other drug use [26, 27]. Consequently, the current clinical practice guidelines for medical cannabis for Canadian physicians do not support its use for anxiety and other mental health conditions [28].

The legalization of recreational cannabis in October 2018 has led to an increased interest in medical cannabis use, including its use for anxiety and its “sparing effect” for benzodiazepines (and opioids) [29]. Concurrent with the high prevalence of benzodiazepine prescribing, it is imperative to fully investigate the harms and benefits of medical cannabis in order for clinicians and patients to make the best-informed decisions. Thus, the purpose of this study was to examine the association of medical cannabis authorization on benzodiazepine usage in the Alberta population. We hypothesize that medical cannabis authorization would be associated with a reduction in chronic benzodiazepine use (i..e, reduction in daily dose).

## Methods

### Study design

A matched cohort study with patients on regular benzodiazepine treatment authorized to use medical cannabis and controls who do not have authorization for medical cannabis.

### Data source

The College of Physicians and Surgeons of Alberta provided the medical cannabis patient identifiers as they are the regulatory entity for cannabis authorization in the province. Through the use of unique lifetime personnel health numbers, all patients were linked to the administrative databases of Alberta Health which captures all healthcare utilization for all patients in the province of Alberta as part of the universal health insurance plan for residents. These databases included provincial health care registry, vital statistics, all inpatient hospitalizations, ambulatory emergency department visits, all community pharmacy drug dispensations, and physician claims data, providing at least one-year of longitudinal follow-up data following the index date for both patients authorized to access medical cannabis and high dimensional propensity score matched controls as outlined below. All data was de-identified prior to its released to the researchers.

### Population

#### Inclusion criteria

The exposed group were all patients prescribed regular benzodiazepine treatment and authorized for medical cannabis in Alberta between March 31, 2013 and March 31, 2021. Medical cannabis authorization in Canada is defined as a patient being granted authorization by their healthcare provider to access cannabis for medical purpose (i.e., patients self-treating with cannabis for medicinal purposes were not included). Participants were of 18 years of age and older, any sex, ethnicity, and socioeconomic status who received authorization for medical cannabis for any indication (acute and chronic). The index date for each patient was the first recorded date of medical cannabis authorization at the clinics. Regular benzodiazepine treatment was defined as those who had:

1. Benzodiazepine dispensation within 30 days prior to the first date of medical cannabis authorization (index date); and
2. A total of 120 or more cumulative calendar days of benzodiazepines prescriptions based on days supply; or 10 or more dispensations in the year prior index date [29].

The unexposed control group met the same above criteria as the exposed group, with the exception of not having medical cannabis authorization. The index dates of the unexposed group were randomly assigned based on the distribution of exposed group’s index dates.

#### Exclusion criteria

All patients who were not eligible to receive health benefits in Alberta were excluded (e.g., out of province patients). In addition, to ensure sufficient follow-up time to assess the effects of medical cannabis authorization on regular benzodiazepine use, all patients had to have at least 1 year follow-up time, maintain eligibility for Alberta Health benefits (i.e., did not move out of province) and did not die within 1 year after the index date. Any patients not meeting these criteria were excluded.

#### Propensity score matched controls

To construct the propensity score, high-dimensional propensity score (HDPS) was used as the approach is known to balance the potential confounders (baseline covariates) and thus, significantly reduce bias by confounding. HDPS can also reduce confounding by some unmeasured characteristics depending on the underlying correlations with known variables. All patients authorized for medical cannabis (*n* = 9690) were matched with one unique unexposed control group patient using the high dimensional propensity score (HDPS) matching. We selected the matched control for each cannabis user using the nearest neighbour approach with 1: 1 ratio and a caliper set at 0.2. Based on this algorithm, we were successful in identifying 1 control for each cannabis users. Balance in confounders was fully assessed, and no imbalances were noted (all standardized mean differences < 0.1 as recommended). Variables incorporated into the HDPS matching method included: sex, age, year of index date (categorical), baseline benzodiazepine duration, social deprivation index, age group, CNS medications, living area (rural/urban), comorbidities associated with cannabis use, and all healthcare resource utilization variables (all within the year prior to the index date). This includes healthcare utilization (all hospitalizations with up to 10 CCI (Canadian Classification of Health Interventions) procedure codes and 25 diagnostic ICD-10 codes, emergency visit with up to 10 ICD-10 diagnostic code, physician claims with CCI procedure code and with up to 3 ICD-9 diagnostic codes) and all prescription drugs dispensed to a patient at baseline. Notably, the entire healthcare dataset reported greater than 1000 different variables and categories which were included in the HDPS. The HDPS matching technique used the SAS packages proposed by Rassen et al. [30] and Schneeweiss et al. [31].

#### Outcomes

All benzodiazepine doses were converted into a defined daily dose (DDD) based on the drug’s day supply, dispensation amount, and strength. Using DDD allowed us to estimate the average maintenance dose per day for the different types of benzodiazepines. To standardized the DDD, as benzodiazepines can be short- or long-acting, the strength of each benzodiazepine was then converted to a diazepam equivalence using known pharmacologic conversion factors [1, 32]. The primary outcome was the difference in the daily average diazepam equivalence (DDE) between the medically authorized patients and the matched control group in the 12 months prior to index and 12 months following medical cannabis authorization (or equivalent index date for controls).

#### Ethics approval

This study was approved by the University of Alberta Health Research Ethics Board (PRO 00084689). As the study relied on secondary use of de-identified health data (i.e. administrative data), a waiver for informed consent was provided by the Ethics Board.

#### Statistical analysis

All data were expressed descriptively using means (standard deviations [SD]) or count (proportions [%]), as appropriate. To assess the effect of medical cannabis use on DDE, interrupted time series (ITS) analysis was used to assess the change in the trend of diazepam equivalence in the 12 months before and 12 months after the authorization of medical cannabis (or pseudo index for matched controls). ITS is a quasi-experimental design that allows comparison of trends in an outcome before and after an intervention [33, 34]. This specific analysis was chosen for its effectiveness in clear differentiation between population-level health pre-intervention and post-intervention periods. The controlled ITS employed in this study included an additional control series to account for temporal changes that may have occurred within the population. Controlled ITS has been shown to provide similar results as those observed in RCTs [35, 36], which highlights the validity of the approach [37, 38].

DDE was assessed in 30-day windows for each patient (i.e., month-to-month average diazepam equivalence). The absolute effect of medical cannabis authorization on average monthly DDE was calculated, which summarizes both the immediate level change (i.e. within the first month following the index date) and change in trend over the 1 year follow-up period with the multivariate delta method used to construct 95% confidence intervals around the estimate [39].

#### Sensitivity and stratification analysis

Further stratification was conducted on both authorized medical cannabis patients (*n* = 9690) and matched controls in 4 subgroups according to baseline DDE as any change in DDE would be expected to be affected by the initial starting DDE:

1. DDE ≤ 5 mg
2. DDE between 5 and 10 mg
3. DDE between 10 and 15 mg
4. DDE > 15 mg

## Results

**Fig. 1:**
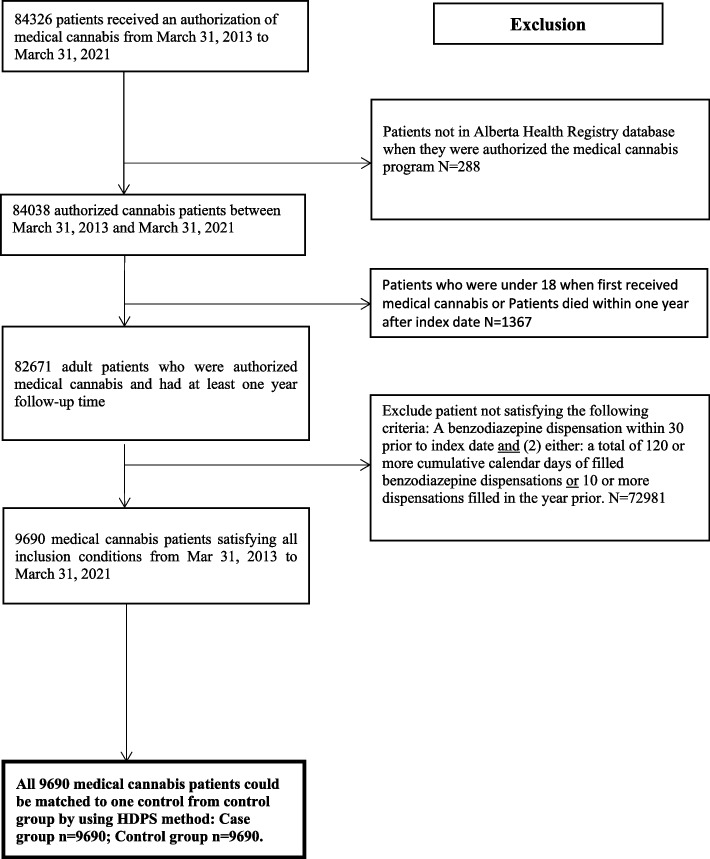
Selection of study population

In total, 9690 medically authorized cannabis patients and 123,899 eligible controls were identified (Fig. 1). All 9690 patients were matched to one control, and following HDPS matching, all covariates were well balanced after matching between the groups (standardized difference < 10%; a threshold recommended for declaring imbalance in pharmacoepidemiologic research) [40] (Table 1).

**Table 1:** Baseline characteristics of those authorized for medical cannabis and matched controls (*n* = 19,380)

Characteristic	Controls (*N* = 9690)	Authorized for medical cannabis (*N* = 9690)	*p*-value	Standardized Difference
Age, years, mean (SD)	53.7 (14.5)	54.4 (13.9)	0.06	0.04815
Female, n (%)	6147 (63.4%)	6170 (63.7%)	0.74	0.04815
Rural, n (%)	1235 (12.8%)	1251 (12.9%)	0.75	0.00494
Age group				
< 35	1060 (10.9%)	1040 (10.7%)	0.66	−0.00664
35–50	2426 (25.0%)	2346 (24.2%)	0.18	−0.01916
50–65	4046 (41.8%)	3907 (40.3%)	0.05	−0.02916
> 65	2158 (22.3%)	2397 (24.7%)	< 0.01	0.05819
Social material deprivation index:				
1	1889 (19.5%)	1768 (18.3%)	0.03	−0.03192
2	1848 (19.1%)	1921 (19.8%)	0.19	0.01903
3	1693 (17.5%)	1788 (18.5%)	0.08	0.02554
4	2033 (21.0%)	2127 (21.9%)	0.10	0.02363
5	2227 (23.0%)	2086 (21.5%)	0.02	−0.03499
**Medications**				
Benzo durations, mean (SD)	0.82 (0.21)	0.82 (0.21)	0.87	0.00005
Antiepileptics	5140 (53.0%)	5038 (52.0%)	0.14	−0.02108
Anti-Parkinson drugs	387 (4%)	394 (4.1%)	0.80	0.00367
Psycholeptics	9153 (94.5%)	9155 (94.5%)	0.97	0.00090
Psychoanaleptics	7089 (73.2%)	6934 (71.6%)	0.01	−0.03577
Other nervous system drugs	1146 (11.8%)	1086 (11.2%)	0.18	−0.01940
**Comorbidities**				
Neoplasms, n (%)	2104 (21.7%)	2241 (23.1%)	0.02	0.03391
Diabetes, n (%)	1387 (14.3%)	1239 (12.8%)	0.01	−0.04464
Mental Disorder, n (%)	8002 (82.6%)	7910 (81.6%)	0.08	−0.02477
Nerve System Disease, n (%)	2449 (25.3%)	2704 (27.9%)	< 0.01	0.05959
Chronic Obstructive Pulmonary Disease, n (%)	1813 (18.7%)	1723 (17.8%)	0.09	−0.02477
Colitis, n (%)	197 (2.0%)	259 (2.7%)	< 0.01	0.04222
Diseases of the Musculoskeletal System and Connective Tissue, n (%)	6863 (70.8%)	7221 (74.5%)	< 0.01	0.08298
Injury and Poisoning, n (%)	2789 (28.8%)	2616 (27.0%)	0.01	−0.03982
**Healthcare Utilization**				
Patients with at least one inpatient hospitalization, n (%)	1654 (17.1%)	1621 (16.7%)	0.54	−0.00909
Patients with at least five outpatient visits, n (%)	3893 (40.2%)	3824 (39.5%)	0.32	−0.01455
Patients with at least five distinct drug class dispensations, n (%)	9376 (96.8%)	9331 (96.3%)	0.08	−0.02537

**Fig. 2:**
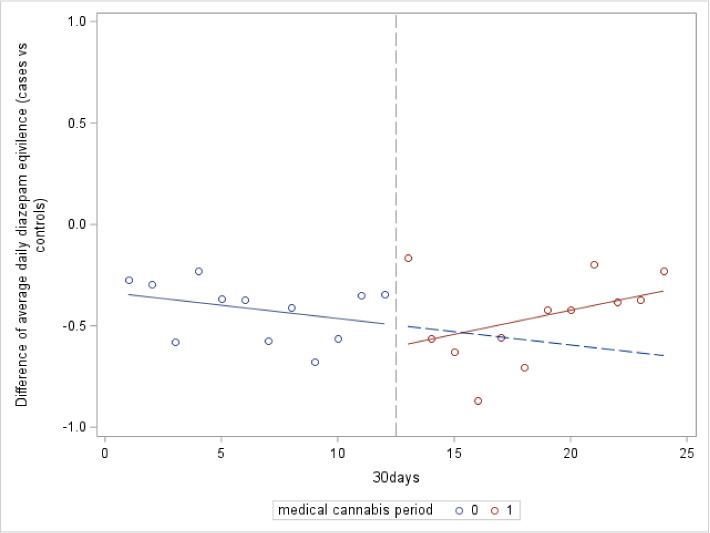
Difference in average daily diazepam equivalents per patient medically authorized cannabis users (*n* = 9690) vs controls (*n* = 9690)

Over the 1-year follow up period after medical cannabis authorization, there was no overall change in the DDE use in authorized medical cannabis patients compared to matched controls (− 0.08 DDE, 95% CI: − 0.41 to 0.24). Additionally, there was no effect in the month-to-month change in average DDE after cannabis authorization (0.04 DDE, 95% CI: − 0.01 to 0.09). When both the initial change and the longer month to month change were combined, no overall effect was observed in the absolute difference in the total monthly DDE (0.32, 95% CI: − 0.23 to 0.87) per patient between cases and controls (Table 2; Fig. 2). Assessments on the type of benzodiazepines dispensed also indicated there was no change in the most frequently used benzodiazepines used before and after cannabis authorization (*p* > 0.05).

**Table 2:** Interrupted time series of average daily diazepam equivalence differences per patient in medically authorized cannabis users (*n* = 9690) vs controls (*n* = 9690)

Variable	Daily diazepam equivalence difference (95% CI) ^a^
Pre-incentive trend ^c^	− 0.02 (− 0.05 to 0.02)
Level change after medical cannabis authorization ^d^	−0.08 (− 0.41 to 0.24)
Trend change after medical cannabis authorization ^e^	0.04 (− 0.01 to 0.09)
Overall absolute effect after medical cannabis authorization ^f^	0.32 (−0.23 to 0.87)

^a^All reported values indicate the average difference in average daily diazepam equivalence per patient in those who received a medical cannabis authorization compared to controls

^c^Rate of change in the outcome over time prior to medical cannabis authorization

^d^Immediate change in outcome following medical cannabis authorization

^e^Month to month change in average daily diazepam equivalence or slope after medical cannabis authorization, relative to the pre-incentive difference in trend

^f^The overall absolute effect after medical cannabis authorization is the absolute difference in the average daily diazepam equivalence over the 12 months pre- and 12 months post-medical cannabis authorization period, compared to the counterfactual difference in trends had medical cannabis authorization not occurred (i.e. pre-incentive difference in trends projected forward)

### Sensitivity analyses results

Among patients consuming ≤5 mg baseline DDE, there was no change immediately after medical cannabis authorization compared to controls (level change, − 0.04 DDE, 95% CI: − 0.12 to 0.03) per patient as well as in the month-to-month trend change (0.002 DDE, 95% CI: − 0.009 to 0.12) per patient was noted. Overall, there was no change in the absolute difference in the total monthly DDE among medically authorized patients after accounting for both immediate and overall trend during follow-up (− 0.02 DDE, 95% CI: − 0.16 to 0.11) compared to controls (Table 3). Results observed among patients consuming 5–10 DDE, 10–15 mg DDE, and > 15 mg DDE were similar with no immediate change, month-to-month trend change, nor overall absolute difference in the total monthly DDE per patient (Table 3).

**Table 3:** Interrupted time series estimates of daily diazepam equivalence differences within baseline diazepam equivalence subgroups per patient in medically authorized cannabis users (*n* = 9690) vs controls (*n* = 9690)

Variable	Daily diazepam equivalence difference in those <=5 mg baseline Daily diazepam equivalence (*n* = 5443 cannabis patients and 5402 controls) ^a^	Daily diazepam equivalence difference in those 5–10 mg baseline Daily diazepam equivalence (*n* = 842 cannabis patients and 812 controls) ^a^	Daily diazepam equivalence difference in those 10–15 mg baseline Daily diazepam equivalence (*n* = 651 cannabis patients and 626 controls) ^a^	Daily diazepam equivalence difference in those > 15 mg baseline Daily diazepam equivalence (*n* = 2754 cannabis patients and 2850 controls) ^a^
Pre-incentive trend ^c^	−0.0002 (− 0.008 to 0.007)	−0.06 (− 0.13 to − 0.001)	−0.03 (− 0.13 to 0.08)	−0.02 (− 0.12 to 0.09)
Level change after medical cannabis authorization ^d^	− 0.04 (− 0.12 to 0.03)	−0.16 (− 0.74 to 0.42)	−0.65 (−1.58 to 0.29)	−0.17 (−1.17 to 0.82)
Trend change after medical cannabis authorization ^e^	0.002 (−0.009 to 0.012)	0.06 (− 0.03 to 0.15)	0.08 (− 0.09 to 0.25)	0.07 (− 0.08 to 0.22)
Overall absolute effect after medical cannabis authorization ^f^	−0.02 (− 0.16 to 0.11)	0.48 (− 0.48 to 1.45)	0.69 (− 0.78 to 2.17)	0.48 (− 1.22 to 2.19)

^a^All reported values indicate the average difference in average daily diazepam equivalence per patient in those who received a medical cannabis authorization compared to controls

^c^Rate of change in the outcome over time prior to medical cannabis authorization

^d^Immediate change in outcome following medical cannabis authorization

^e^Month to Month change in average daily diazepam equivalence or slope after medical cannabis authorization, relative to the pre-incentive difference in trend

^f^The overall absolute effect after medical cannabis authorization is the absolute difference in the average daily diazepam equivalence over the 6 month pre- and 12 months post-medical cannabis authorization period, compared to the counterfactual difference in trends had medical cannabis authorization not occurred (i.e. pre-incentive difference in trends projected forward)

## Discussion

In this retrospective, observational, population-based study, short-term analysis demonstrated that medical cannabis authorization had little to no effect on benzodiazepine usage among patients prescribed regular benzodiazepine treatment in Alberta, Canada. There were no differences observed across a wide range of initial DDE at baseline. Notably, there were no statistically significant differences when comparing different categories of benzodiazepine DDE groups and any small increases or decreases are likely clinically inconsequential. Collectively, these results may suggest that for the majority of patients on regular benzodiazepine treatment, medical cannabis use is unlikely to alter future benzodiazepine use.

Regarding the literature on benzodiazepine use, more studies are now supporting the role of medical cannabis as an adjunct to benzodiazepine tapering and cessation. Also known as a “sparing effect treatment,” recent studies are reporting that concurrent use with medical cannabis can reduce future benzodiazepine use [41]. Particularly, Purcell et. al’s work found that 45.2% patients successfully discontinued their pre-existing benzodiazepine therapy over 2 months of medical cannabis therapy [42]. However, it is important to note that in this study, the sample size was much smaller and had more frequent follow-ups with physicians compared to the larger sample in our study. Additionally, another study [43] on perioperative cannabis use on surgical patients showed that concurrent use decreased overall benzodiazepine and opioid use. Although our study showed no effect, subgroup effects may still exist where certain patients on benzodiazepine treatment were able to successfully decrease their benzodiazepine dosage through medical cannabis use.

A significant strength of this study is that it is currently, to our knowledge, the largest and longest population-based study of medical cannabis users in Canada. It uses robust measures to track medical cannabis authorization and current benzodiazepine treatment. However, our study is not without limitations. It is an observational study, which can be prone to potential spectrum bias as our cohort of patients were those who individually sought medical cannabis authorization. Secondly, there is a lack of insight into the patient’s adherence to their authorized medical cannabis treatment. The cannabis may have been taken differently than as indicated and/or alternative therapies may have been used to circumvent benzodiazepine usage. Notably, we do not know whether patients were using cannabis prior to authorization. Further, we do not know whether legalization of cannabis during this period impacted the number of patients seeking medical cannabis authorization. In this case, we may expect non-differential misclassification of the control group due to increased widespread cannabis legalization after 2018. For example, some controls may report not having been medically authorized for cannabis, but after legalization of cannabis, were accessing recreational cannabis via storefront for other reasons. The extent of this occurrence is unknown but notably the majority of patients in our study were derived prior to the legalization change in 2018 (i.e., 2013–2018). Furthermore, given the wide variability of medical cannabis products available in Canada, we could not analyze specific strains, modes of consumption, or dosing regimens in this study. All cannabis products were treated as equals, despite there being known clinical difference between products and regimens. Our study is therefore limited by the lack of these clinical details of medical cannabis, in addition to the lack of concomitant use with other non-prescription benzodiazepines agents.

## Conclusion

This study found that medical cannabis authorization had minimal effects on benzodiazepine use. Although the clinical importance of medical cannabis as a benzodiazepine sparing treatment is unclear, our findings contribute ongoing evidence for clinicians regarding the association between medical cannabis on benzodiazepine use.

## Data Availability

The data that support the findings of this study are available from College of Physicians & Surgeons of Alberta (CPSA) and the Alberta SPOR SUPPORT Unit (https://absporu.ca) but restrictions apply to the availability of these data, which were used under license for the current study, and so are not publicly available. We had full permission to use this data, however, specific restrictions do apply to the public availability of these data, which are under data access agreements for the current study.
